# Functionalization of a Cortical Membrane with a Photodynamic Protocol

**DOI:** 10.3390/jfb14030133

**Published:** 2023-02-27

**Authors:** Tania Vanessa Pierfelice, Emira D’Amico, Simonetta D’Ercole, Stefania Lepore, Adriano Piattelli, Antonio Barone, Giovanna Iezzi, Morena Petrini

**Affiliations:** 1 Department of Medical, Oral and Biotechnological Sciences, University “G. d’Annunzio” of Chieti-Pescara, Via dei Vestini 31, 66013 Chieti, Italy; 2 School of Dentistry, Saint Camillus International University of Health and Medical Sciences, Via di Sant’Alessandro 8, 00131 Rome, Italy; 3 Facultad de Medicina, UCAM Universidad Catolica San Antonio de Murcia, 30107 Murcia, Spain; 4 Department of Surgical, Medical, Molecular Pathologies and of the Critical Needs, School of Dentistry, University of Pisa, 56100 Pisa, Italy; 5 Complex Unit of Stomatology and Oral Surgery, University-Hospital of Pisa, 56100 Pisa, Italy; 6 Unit of Oral Surgery and Implantology, University Hospitals of Geneva, University of Geneva, 1205 Geneva, Switzerland

**Keywords:** lamina cortical membrane, photodynamic therapy, osteoblasts, 5-aminolevulinic acid, LED, ALP, calcium deposition, GBR

## Abstract

Guided bone regeneration (GBR) comprehends the application of membranes to drive bone healing and to exclude non-osteogenic tissues from interfering with bone regeneration. However, the membranes may be exposed to bacterial attack, with the risk of failure of the GBR. Recently, an antibacterial photodynamic protocol (ALAD-PDT) based on a gel with 5% 5-aminolevulinic acid incubated for 45 min and irradiated for 7 min by a LED light at 630 nm, also showed a pro-proliferative effect on human fibroblasts and osteoblasts. The present study hypothesized that the functionalization of a porcine cortical membrane (soft-curved lamina, OsteoBiol) with ALAD-PDT might promote its osteoconductive properties. TEST 1 aimed to verify the response of osteoblasts seeded on lamina with respect to the plate surface (CTRL). TEST 2 aimed to investigate the effects of ALAD-PDT on the osteoblasts cultured on the lamina. SEM analyses were performed to study the topographical characteristics of the membrane surface, the adhesion, and the morphology of cells at 3 days. The viability was assessed at 3 days, the ALP activity at 7 days, and calcium deposition at 14 days. Results showed the porous surface of the lamina and the increase in cell attachment of osteoblasts with respect to controls. The proliferation, the ALP, and bone mineralization activity of osteoblasts seeded on lamina resulted in being significantly higher (*p* < 0.0001) than controls. Results also showed an additional significative enhancement (*p* < 0.0001) in the proliferative rate in ALP and calcium deposition after applying ALAD-PDT. In conclusion, the functionalization of the cortical membranes cultured with osteoblasts with the ALAD-PDT improved their osteoconductive properties.

## 1. Introduction

In photodynamic therapy (PDT), an exogenous photosensitizer (PS) is applied locally or systemically, and after a certain period of incubation, it is activated by light irradiation with a specific wavelength [1]. The mechanisms of action of PDT are not fully understood. However, it is known that, in the presence of oxygen inside the target cell, one of the main effects of PS photoactivation is the production of reactive oxygen species (ROS), highly unstable molecules with a cytotoxic effect [2]. PDT, due to its characteristics, is an emerging, non-invasive treatment method in dentistry in which the PS is applied locally, although, in other fields of medicine, the application of PS can also be systemic [3,4]. The efficacy of PDT depends on many factors related to the light and the PS [5]. The applied PS should be the specific target of the light, and it should be able to diffuse in the tissues, permeate them, and selectively accumulate only on pathogenic ones. Indeed, an interesting aspect of PDT is the selective accumulation of the PS in the diseased tissues that permit the elimination of pathological tissues without affecting the surrounding healthy ones [2]. The light wavelength is a fundamental factor because it should be specific to the PS applied. It also influences the light penetration depth: shorter wavelengths cannot penetrate deeper tissues. The 5 delta-aminolevulinic acid (5-ALA) is the precursor of the PS protoporphyrin IX (PpIX), a molecule belonging to the pathway that culminates with the production of the heme group. Mammalian cells convert PpIX into heme by the enzyme ferrochelatase [6,7,8,9]. On the contrary, bacteria and neoplastic cells characterized by a high turnover and high deficiency of this enzyme, after the adsorption of 5-ala, can accumulate high quantities of PpIX and, after light irradiation, an intense ROS production permits their eradication [9,10]. The 5-ala has been successfully applied in dermatology and for cancer diagnosis and treatment in the last 30 years; however, concerning the antimicrobial activity, the effect was not constant, especially against Gram-negative bacteria. Recently, a novel gel (ALAD) containing 5% of 5-ALA with pH 3.5 in a patented formulation has been produced especially for the treatment of periodontitis and peri-implantitis. ALAD showed in vitro activity against both Gram-positive and Gram-negative bacteria [11,12,13] and encouraged preliminary in vivo results [14]. The antibacterial effect was obtained by photoactivation of ALAD with different light emitters of red light at 630 nm, such as single-led TL-01, or full-mouth multi-LED Tl-03, or optic fibers for endodontic uses [15,16]. Some authors have hypothesized that the antifungal activity of the ALAD-PDT protocol shown against Candida albicans could be not only the result of ROS production but also the consequence of other properties intrinsic to the ALAD gel formulation, such as the low pH. With respect to other photodynamic therapy, the novelty of ALAD-PDT is that it was also able to promote fibroblasts and osteoblasts proliferation and osteogenic bone marker increments [17,18]. This effect opens up a possible clinical use of the ALAD-PDT protocol during guided bone regeneration (GBR) as an adjunctive aid for both antibacterial activity and accelerated healing. The GBR is usually carried out by the application of bioresorbable or non-resorbable membranes that serve to create a suitable environment to drive the bone healing and remodeling processes. During this procedure, a membrane is used to stabilize the coagulum, maintain the space, and direct the cellular proliferation and differentiation of osteoblastic cells by blocking the epithelial cells that are characterized by higher proliferation, thus promoting the growth of osteoblasts [19]. Recently, a new generation of naturally derived membranes made of cortical bone of heterologous origin has been produced. These types of cortical membranes are characterized by a semi-rigid and shapeable consistency to fit the alveolar bone morphology. Thus, the first objective of this study was to evaluate, in vitro, the human oral osteoblasts (HOBs) response to a porcine cortical membrane (the soft-curved lamina, OsteoBiol, Tecnoss ^®^ s.r.l., Giaveno, Italy). The first part of this experimentation aimed to verify that the membrane was osteoconductive, comparing the lamina with the plate surface (CTRL). However, the membranes usually applied during GBR may be exposed to the oral cavity and undergo a bacterial attack, causing contamination of bone graft materials and difficulty in bone regeneration [20]. Currently, to overcome these complications, many studies are focusing on the coating of membranes with some bioactive materials, such as hydroxyapatite and/or alginate/chitosan, to improve both osteoinductive and antibacterial properties [19,21]. Photodynamic therapy has evolved through the years with developments in photosensitizers and more complex methodologies resulting in alternative treatments employed as cancer therapy [22], alleviation of autoimmune disease complications [23], and control or elimination of viral, fungal and bacterial infections in both planktonic and biofilm forms [24]. However, few studies reported the application of photodynamic treatments during GBR procedures [25,26]. In those studies, all photodynamic protocols were applied as antimicrobial treatments and occurred previously to guide bone regeneration, which was carried out using xenogenic bone and resorbable collagen membranes. In view of our recent in vitro studies concerning the pro-proliferative effects of ALAD-PDT protocol on fibroblasts and osteoblasts [17,18], the present study proposed innovative employment of the photodynamic treatment by hypothesizing that the application of ALAD-PDT on curved lamina could promote its osteoconductive properties to achieve bone gain during GBR. Thus, the second objective was to functionalize the curved lamina with the application of the ALAD-PDT protocol in order to investigate the effects of ALAD-PDT on the adhesion, the growth, and the mineralization activity of HOBs seeded on the lamina.

## 2. Materials and Methods

### 2.1. Materials

#### 2.1.1. Membrane

**Figure 1:**
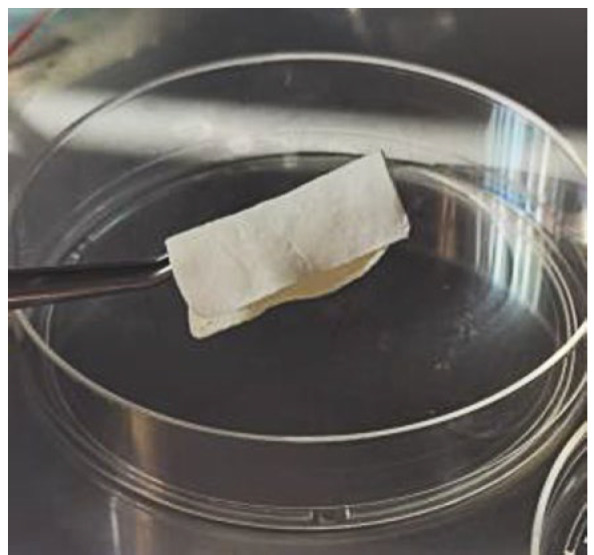
Cortical bone of heterologous origin OsteoBiol^®^ lamina.

The membrane (OsteoBiol^®^ lamina, Tecnoss ^®^ s.r.l., Giaveno, Italy) was naturally derived and was made of cortical collagenated bone of heterologous origin, characterized by a semi-rigid consistency and a shape to fit the alveolar bone morphology (Figure 1).

For in vitro test, it was cut in squares of 5 × 5 mm in sterilized conditions.

#### 2.1.2. ALAD-PDT Protocol

**Figure 2:**
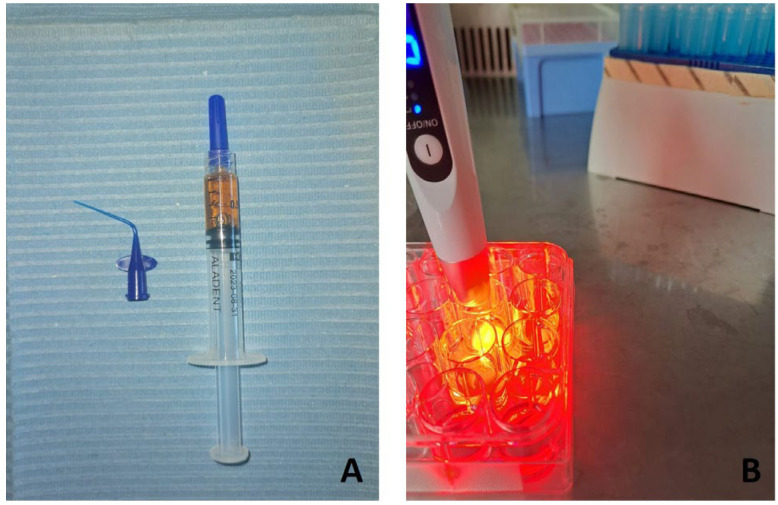
ALAD-PDT protocol consists of a gel containing 5% of delta-aminolevulinic acid distributed in a disposable syringe (**A**) and a LED device TL-01 as red-light source (**B**).

In this study, a thermosensitive gel containing 5% of 5-delta-aminolevulinic acid (ALAD), commercialized as ALADENT (AlphaStrumenti s.r.l, Melzo-MI, Italy) has been utilized. The medical device, covered by a patent (PCT/IB2018/060368, 12.19.2018), was a thermo-setting product that remains liquid at temperatures below 28 °C and becomes gel at higher temperatures (Figure 2A).

The light source used is AlGaAs power light-emitting diode (LED) device TL-01(ALPHA Strumenti, Italy). TL-01 is a single LED emitter, meaning that the light beam is derived from a glass point having a 6 mm diameter. During the tests, the LED source stood in perpendicular, with respect to the wells, and the illumination arrived from the top of the plates at 0.5 mm of distance (Figure 2B). The illumination setting was 380 mW/cm^2^ power, 630 nm irradiation wavelength, and 23 J/cm^2^ energy density.

### 2.2. Experimental Design

To address the objectives, TEST 1 and TEST 2 were carried out as follows:

- In TEST 1, the response of human oral osteoblasts (HOBs) was evaluated for lamina compared to ones seeded on a well/plate. Two groups were compared, HOBs seeded on the plate as control (CTRL) and HOBs seeded on the membrane (lamina). SEM analyses were performed to study the topographical characteristics of the membrane surface and the adhesion and morphology of cells at 3 days. The viability was assessed at 3 days by MTT assay, the ALP activity was investigated at 7 days by ALP enzymatic assay, and calcium deposition at 14 days was assessed by Alizarin Red staining and by the measurement of absorbance;
- In TEST 2, the activity of HOBs seeded on the lamina was compared to the response of HOBs seeded on the lamina surface and then subjected to the ALAD-PDT protocol.

Four groups were compared:

- HOBs seeded on the membrane (lamina);
- HOBs seeded on the membrane and treated with ALAD gel for 45 min (ALAD);
- HOBs cultured on the membrane and irradiated with 630 nm LED for 7 min (PDT);
- HOBs cultured on the membrane, treated with ALAD gel, and irradiated with 630 nm LED for 7 min (ALAD-PDT).

SEM analyses were performed to study the adhesion and to observe the morphology of cells at 3 days. The viability was assessed at 3 days by MTT assay, the ALP activity was investigated at 7 days by ALP enzymatic assay. Calcium deposition at 14 days was assessed by Alizarin Red staining and by the measurement of absorbance.

All experiments were performed in triplicate.

### 2.3. Characterization of the Lamina

The cortical bone lamina topography was characterized via a scanning electron microscope (SEM). First, membranes were fixed by 2.5% glutaraldehyde for 1 h, followed by dehydration using sequential concentrations of ethanol (40, 50, 70, 95, and 100°). Then, they were gold-sputtered and observed with SEM (Philips XL20; Philips Inc., Eindhoven, The Netherlands) at 15 kV. The images were taken at 1000×.

### 2.4. Biological In Vitro Tests

#### 2.4.1. Cell Culture

Human oral osteoblasts (HOBs) were obtained from bone biopsies of *n* = 12 patients undergoing the surgical removal of lower third molars at the dental clinic of the G. D’Annunzio University (Ethical Committee reference numbers: BONEISTO N. 22-10.07.2021). In brief, bone fragments were subjected to three enzymatic digestions at 37 °C using collagenase type 1A (Sigma–Aldrich, St. Louis, MO, USA) and trypsin-EDTA 0.25% (Corning, New York, NY, USA). After each digestion, this solution was centrifuged at 1200 rpm for 10 min, and the pellet obtained was transferred into a T25 culture flask with low-glucose (1 g/L) DMEM supplemented with 10% FBS (SIAL, Rome, Italy), 1% antibiotics (100 µg/mL^−1^ streptomycin and 100 IU/mL^−1^ penicillin), and 1% L-glutamine (Corning) at 5% CO_2_ and 37 °C. The medium has been changed every 4–5 days. For tests, HOBs were used from the third and fifth passages after seeding on the top of the lamina at different density and timing points.

#### 2.4.2. MTT Assay

To assess the viability of cells cultured on the membrane, an MTT assay (Sigma–Aldrich) was performed. The 10^4^ cells/membranes were cultured on the top of the lamina in a 96-well plate containing a final volume of 100 µL/well and were incubated at 37 °C. After 3 days of incubation, 10 µL of MTT solution was added per well to achieve a final concentration of 0.5 mg/mL and incubated for 4 h at 37 °C. The quantity of formazan was measured by recording changes in absorbance. The absorbance was read at 650 nm by a microplate reader (Synergy H1 Hybrid BioTek Instruments, Winooski, VT, USA) at 650 nm wavelength. The proliferation levels were expressed in the form of percentages and were calculated with respect to control (CTRL).

#### 2.4.3. Cell Adhesion and Morphology

HOBs at the density of 10^4^ cells/membrane were seeded on the membrane for 3 days. Then, cells were fixed with 2.5% glutaraldehyde for 1 h and were dehydrated using increasing concentrations of ethanol. Before the observation with SEM (Philips XL20; Philips Inc., Eindhoven, The Netherlands), the specimens were gold-sputtered. The images were taken at 1000× and 3000×.

#### 2.4.4. ALP Assay

The 5∙10^4^ HOBs/membranes were seeded on the lamina. After 7 days of culture, ALP levels were measured with ALP assay kit colorimetric AB83369 (Abcam Inc, Cambridge, UK). Upon three washings with PBS, cell lysate was obtained from homogenizing cell suspension through a Tissue Rupture device (QIAGEN, Hilden, Germany). This solution was centrifugated at 10,000× *g* for 15 min, and the supernatant was collected. The test was performed according to the manufacturer, and the absorbance was read at 450 nm by a microplate reader (Synergy H1 Hybrid BioTek Instruments, Winooski, VT, USA).

#### 2.4.5. Alizarin Red Staining and Quantification of Calcium Deposition

HOBs, at the density of 5·10^4^ cells/membrane, were cultured for 14 days at 5% CO_2_ and 37 °C. Then, samples were fixed with glutaraldehyde solution at a concentration of 2.5% for 2 h. Alizarin Red staining solution (Sigma–Aldrich) was added for 1 h at room temperature, and the excess dye was removed using deionized water. Images were taken by a camera. In addition, 1 mL of 10% of Cetylpyridinium Chloride (CPC) (Sigma–Aldrich) was added to quantify calcium deposits through the chelation of calcium ions. After 1 h of incubation, the absorbance was measured at 540 nm by a microplate reader (Synergy H1 Hybrid BioTek Instruments).

#### 2.4.6. Statistical Analysis

A software (GraphPad Prism8, GraphPad Software, San Diego, CA, USA) was used for the statistical analysis. Mean values and standard deviations (SDs) were calculated for each group. In TEST 1, the comparison occurred between two groups; thus, the *T*-test has been chosen as the statistical method, while One way ANOVA followed by the Tukey post hoc test was applied as the statistical tool in TEST 2, in which the comparison occurred for more than two groups [27,28]. Differences were considered to be significant when *p* < 0.05.

## 3. Results

### 3.1. Characterization of the Lamina

**Figure 3:**
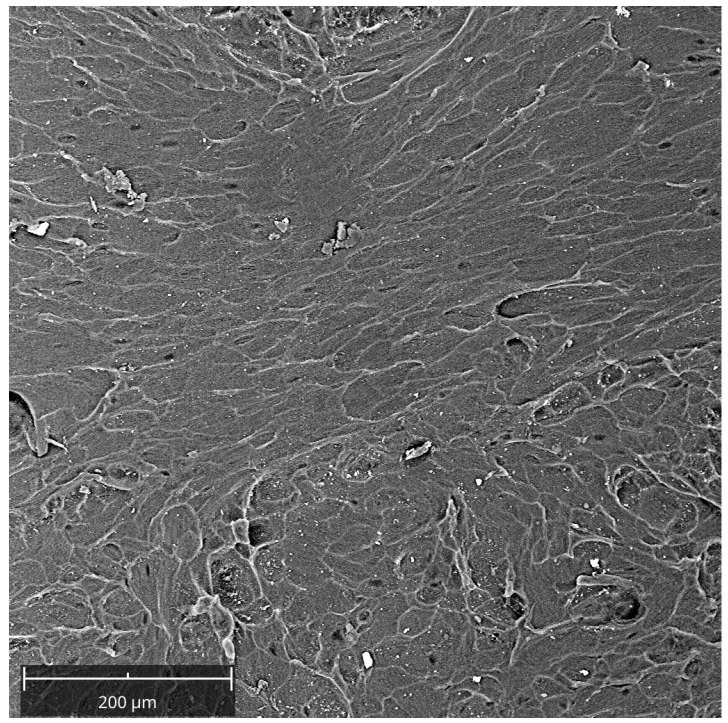
SEM observation of the lamina as manufactured. Mag = 1000×.

The topographical features of the lamina were evaluated with SEM. The observed morphology had a surface with distributed collagen bundles that formed a rough and porous structure (Figure 3).

### 3.2. Biological In Vitro TEST 1

#### 3.2.1. Cell Viability

**Figure 4:**
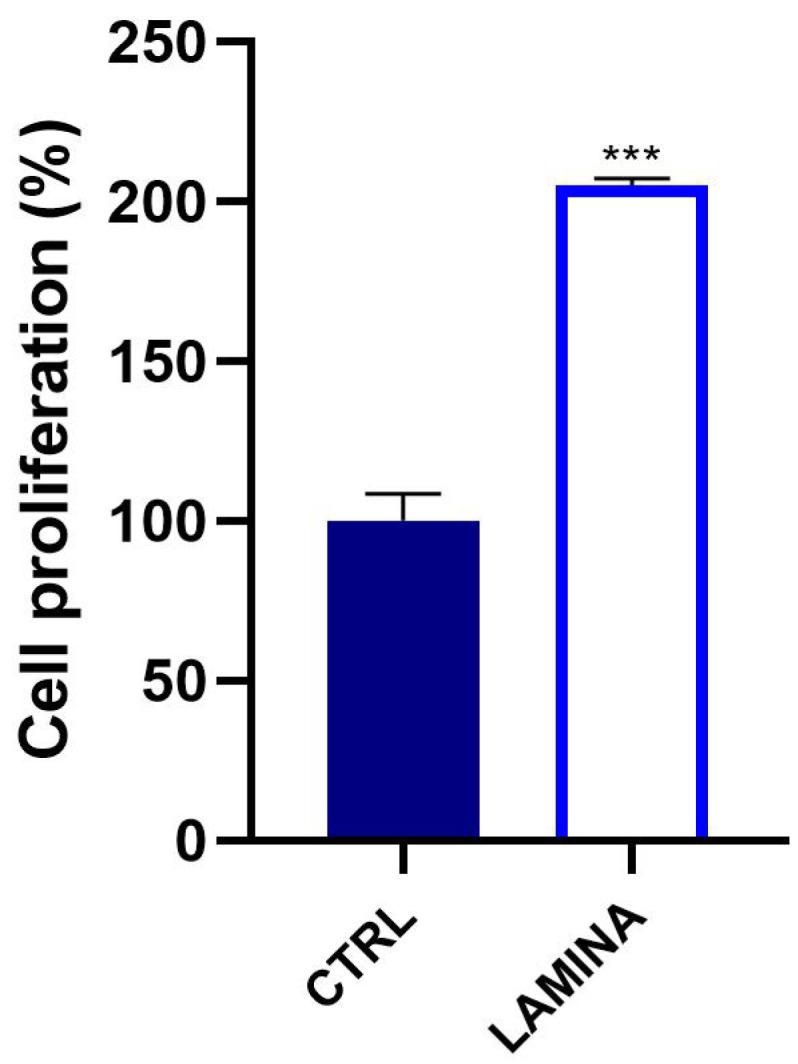
Viability of osteoblasts cultured on the lamina at 3 days. Statistically significant differences were expressed with respect to cells seeded on the well/plate as CTRL (*** *p* < 0.0001).

The viability of osteoblasts cultured on the lamina was evaluated at 3 days. Cells seeded on the lamina showed a statistical enhancement of the growth (*p* < 0.0001) compared to CTRL (Figure 4). The rate of proliferation resulted in being +105.09 ± 2.02%.

#### 3.2.2. Cell Adhesion

**Figure 5:**
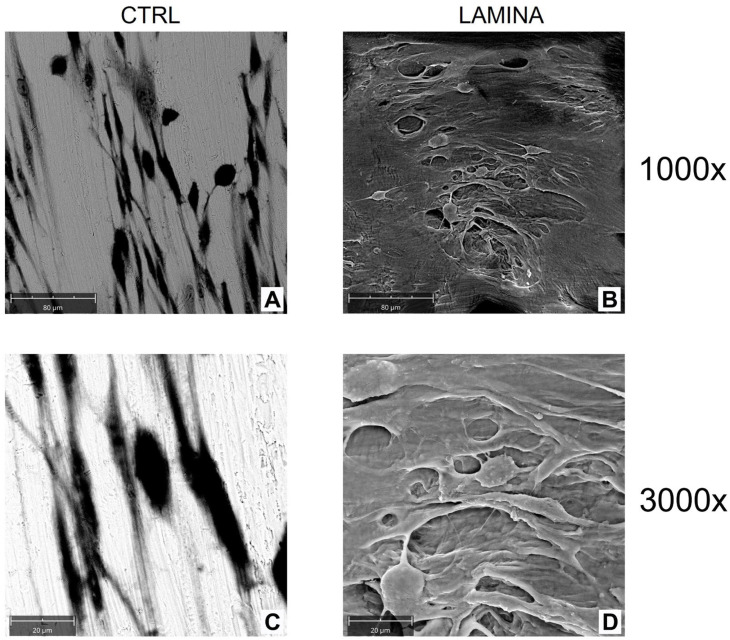
SEM images of oral osteoblasts (HOBs) cultured on the plate (**A**,**C**) and on the lamina cortical membrane (**B**,**D**) at 3 days of culture. Mag = 1000× (**A**,**B**), Mag = 3000× (**C**,**D**).

The adhesion and morphology of HOBs on the lamina were evaluated at SEM after 3 days of culture (Figure 5). HOBs cultured on the lamina appeared more numerous with respect to CTRL. A dense layer of cells was observed, and osteoblasts exhibited a star-shaped morphology (Figure 5B,D). The CTRL showed elongated cells that established a network with each other (Figure 5A,C).

#### 3.2.3. ALP Activity

**Figure 6:**
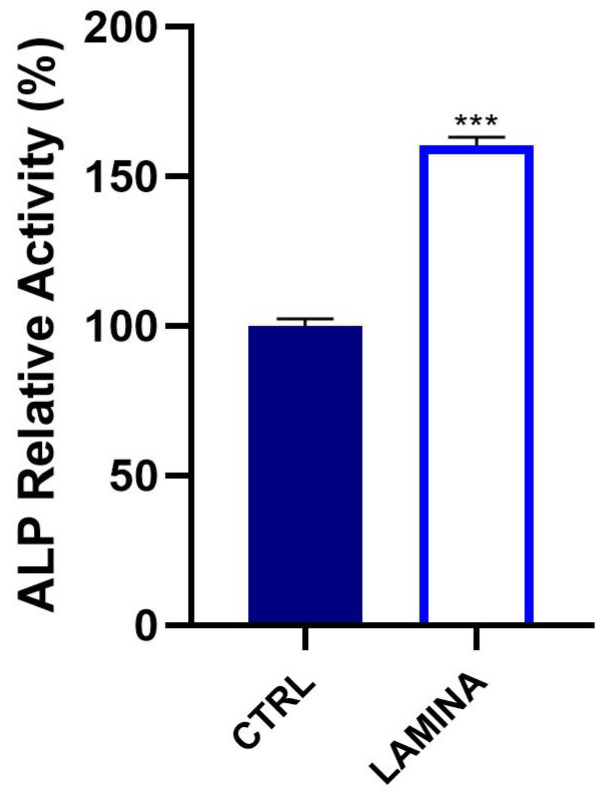
ALP levels of HOBs seeded on the lamina were analyzed at 7 days (*** *p* < 0.0001).

ALP levels of HOBS cultured on the lamina were evaluated after 7 days. They resulted in being significantly increased in cells on the lamina with respect to CTRL (*p* < 0.0001) (Figure 6). The observed increment was +60.47 ± 2.7%.

#### 3.2.4. Mineralization

**Figure 7:**
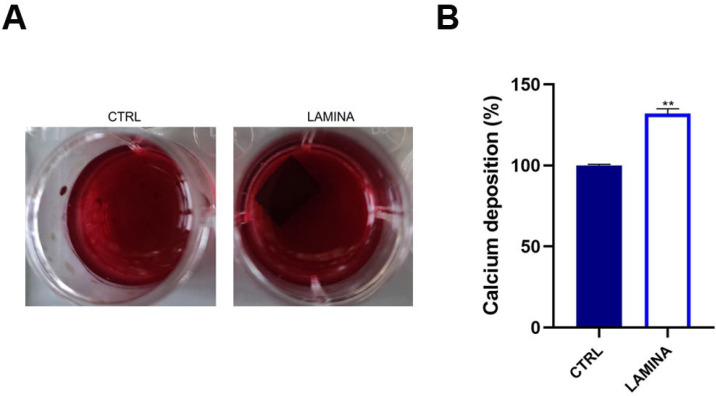
Mineralization of HOBs at 14 days of culture on the lamina (**A**). Qualitative evaluation by Alizarin red staining. (**B**) Quantitative evaluation by CPC (**B**). (** *p* < 0.001).

Alizarin Red staining was used to evaluate qualitatively mineralization, which was quantized by CPC after 14 days of culture (Figure 7). Figure 7A shows brighter red color in lamina than CTRL (Figure 7A). This observation was confirmed by the evaluation of calcium deposits that resulted in being statistically augmented in lamina with respect to CTRL (*p* < 0.001) (Figure 7B).

### 3.3. Biological In Vitro TEST 2

#### 3.3.1. Cell Viability

**Figure 8:**
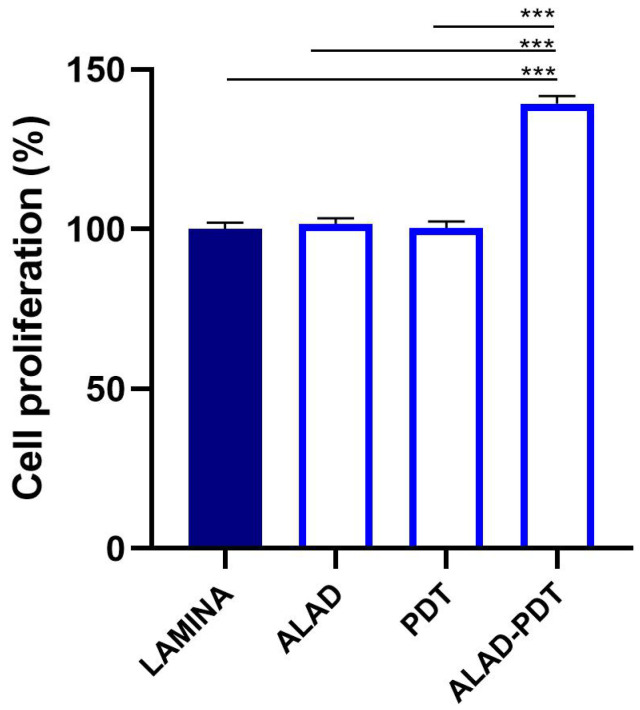
Viability of osteoblasts cultured on the lamina at 3 days and subjected to ALAD-PDT protocol (*** *p* < 0.0001).

The viability of osteoblasts (HOBs) cultured on the lamina was evaluated by MTT assay at 3 days (Figure 8). ALAD-PDT protocol induced a significant enhancement of proliferation compared to the lamina (*p* < 0.0001). A growth rate of +39.29 ± 2.39 was observed. The treatment with ALAD and the irradiation with LED (PDT) did not influence HOBs viability compared to the lamina. Furthermore, cell growth resulted in being statistically augmented from ALAD-PDT with respect to ALAD and PDT.

#### 3.3.2. Adhesion of Osteoblasts on the Lamina

**Figure 9:**
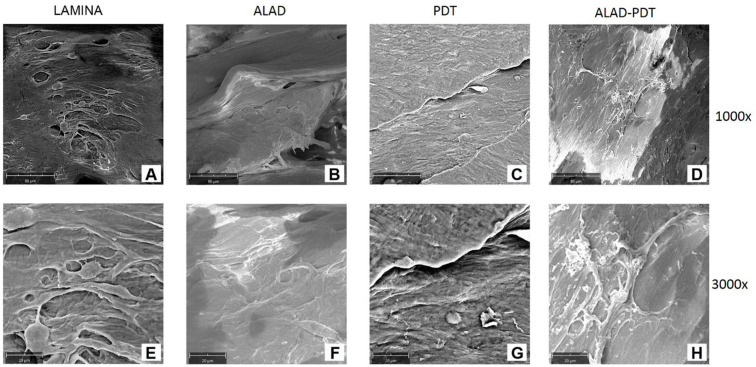
SEM images of oral osteoblasts (HOBs) cultured on lamina cortical membrane and exposed to ALAD-PDT treatment at 3 days of culture. Mag = 1000× (**A**–**D**). Mag = 3000× (**E**–**H**).

Osteoblasts adhesion was evaluated at SEM after 3 days of culture (Figure 9). HOBs exposed to ALAD-PDT protocol colonized the entire surface of the lamina, producing a layer of cells. Thus, at the magnification of 1000×, the detailed morphology of cells was not recognizable (Figure 9D). At higher magnification, the elongated shape of osteoblasts was shown (Figure 9H). Similarly, a cell layer was observed after ALAD treatment (Figure 9B,F). In contrast, when the cells cultured on the lamina were exposed to PDT, a reduced number of osteoblasts was observed (Figure 9C,G). The image of the cells seeded on the lamina revealed the detailed morphology of an osteoblast with a star-like shape (Figure 9A,E).

#### 3.3.3. ALP Activity

**Figure 10:**
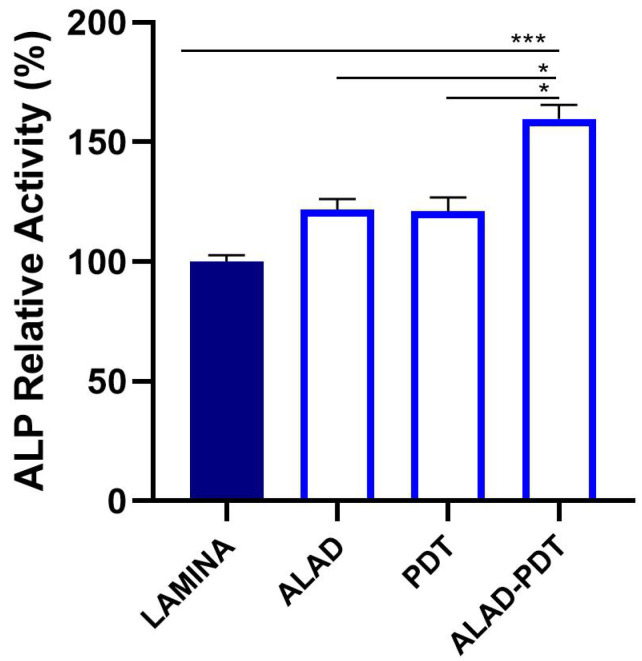
ALP levels of HOBs seeded on the lamina and subjected to ALAD-PDT protocol were analyzed at 7 days (* *p* < 0.05, *** *p* < 0.001).

The ALP activity was analyzed after 7 days of culture (Figure 10). ALAD-PDT induced significant augmented levels of ALP compared to the lamina (*p* < 0.001) with a rate of +59.80 ± 5.81%. ALAD and PDT showed the same ALP levels that resulted in being increased with respect to the lamina. Furthermore, ALAD-PDT statistically stimulated ALP activity compared to ALAD and PDT (*p* < 0.05).

#### 3.3.4. Mineralization

**Figure 11:**
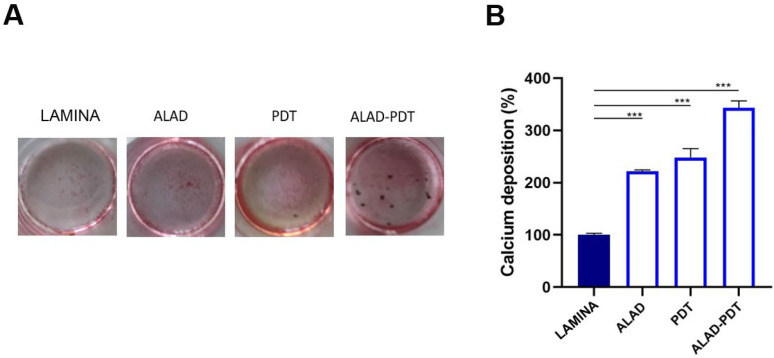
Mineralization of HOBs at 14 days of culture on the lamina and after ALAD-PDT. (**A**) Qualitative evaluation by Alizarin red staining. (**B**) Quantitative evaluation by CPC (**B**). (*** *p* < 0.0001).

Mineralization activity was evaluated qualitatively by Alizarin Red staining (ARS) and quantized by CPC at 14 days (Figure 11). The images of ARS showed the presence of calcified nodules after the ALAD-PDT protocol that appeared brighter red than the lamina. ALAD and PDT conditions exhibited more numerous nodules with respect to lamina but fewer than ALAD-PDT (Figure 11A). These observations were confirmed by the quantization with CPC. Therefore, the highest level of calcium deposition was observed in the ALAD-PDT group with an increment of +243.25 ± 13.30% with respect to the lamina (*p* < 0.0001). Although calcium depositions resulted in an increase in ALAD and PDT compared to the lamina (*p* < 0.0001), they were lower than ALAD-PDT (Figure 11B).

## 4. Discussion

Recently, a new generation of naturally derived membranes made of cortical bone of heterologous origin has been produced to be applied during guided bone regeneration (GBR) techniques. These porcine cortical membranes (soft lamina) are characterized by a semi-rigid curved shape to fit the alveolar bone morphology. This curved flexible lamina is easily adapted by the clinician to the defect morphology, creating, once fixated, a semi-rigid covering to the underlying graft. This property is particularly useful when it is necessary to maintain the graft volume in horizontal augmentation of two wall defects and in lateral sinus lift procedures [29]. Among other characteristics, this type of membrane should meet two fundamental criteria: biocompatibility and promotion of growth of bone-forming cells. Thus, TEST 1 aimed to evaluate, in vitro, the human oral osteoblast (HOB) response to the porcine cortical lamina with respect to the plate surface (CTRL). HOBs were chosen because of their implications in bone physiology and their ability to interact with biomaterials. First, in this study, the lamina topography was observed at SEM because, beyond the adaptability, the membranes should have a porous structure to allow the diffusion of plasma and nutrients and must be a barrier to the invasion of epithelial cells to promote the proliferation of bone-forming cells [19]. SEM observations showed the topographical features of the membrane surface that appeared rough. Lamina membrane was then tested in an in vitro model by seeding and culturing HOBs on their surface, compared to cells seeded on the plastic surface of the well/plate as control. With respect to control, SEM images showed that cells covered the entire lamina surface by creating a dense network, whereas on the plate surface, cells were better distinguishable probably because cells were lower in number than ones on the lamina. This stronger adhesion of cells was in line with the viability assay that revealed a higher significative proliferation rate (+105.09%). Different studies demonstrated a strong correlation between the scaffold surface features and cell attachment and viability [30,31,32]. The lamina membrane used in this study possesses a rough surface with distributed collagen bundles that form a rough and porous structure which promotes osteoblasts attachment and growth. Similarly, lamina significantly enhanced the ALP activity and promoted the calcium deposition of osteoblasts with respect to the ones seeded on the plate. Given that the membranes are employed to favor bone healing and regeneration, recent studies are focusing on the improvement of membrane features by enriching them with some composites, such as chitosan and hydroxyapatite [32]. In a recent study, cortical lamina has been functionalized with graphene oxide to improve the biological properties of the membrane [28]. Considering that an antibacterial photodynamic protocol (ALAD-PDT), in vitro also demonstrated a pro-proliferative effect on fibroblasts and osteoblasts [11,12,13,33], the biological activities of the lamina were also investigated after the application of ALAD-PDT protocol, in TEST 2 [17,18]. The cultured Lamina membranes with HOBs were subjected to the application of ALAD-PDT consisting of incubation for 45 min with a gel containing 5% of 5-aminolevulinic acid and irradiation for 7 min with a red light at 630 nm, to check, whether this photodynamic protocol could improve the early adhesion and proliferation of cells. ALAD-PDT protocol significantly enhanced the proliferation evaluated at 3 days, indicating improved biocompatibility compared to membranes not subjected to photodynamic protocol. SEM has shown that ALAD-PDT allowed the cell to spread throughout the surface of the lamina, promoted their adhesion, and favored the formation of a uniform cell layer. Probably this effect was due to the additive contained in ALAD gel consisting of a poloxamers mixture that permits ALAD to be liquid but to jellify at temperatures higher than 28 °C, so it can easily adhere to oral mucosa and act as glue. The ALAD formulation also avoids the gel being washed out by saliva. As expected, based on our previous studies [17], the application of the ALAD-PDT protocol significantly stimulated the ALP activity at 7 days of culture and significantly enhanced mineralized bone matrix deposition after 14 days, indicating that at mid and late stages, the activity of osteoblasts was promoted. Interestingly, also for the application of LED alone and ALAD gel alone, an increased mineralization activity in HOBs seeded on the lamina cortical membranes was recorded, despite the combination of both as ALAD-PDT protocol stimulating the highest activity. This highlights the importance of following the correct ALAD-PDT protocol, as the photosensitizer needs to be photoactivated by light at specific wavelengths and after a specific incubation time to exert its effects [2]. Even though the effects of photodynamic therapy on cells of healthy tissues are poorly investigated, a recent study also highlighted the absence of adverse effects of a photodynamic protocol on fibroblasts and osteoblasts [34]. Azaripour et al. focused on using methylene blue as the photosensitizer in combination with a soft laser. However, it has been demonstrated that 5-aminolevulinic acid, with respect to methylene blue, is also effective against Gram-negative bacteria [12,34]. Although another study showed that the functionalization of the cortical lamina with graphene oxide stimulated the growth and the osteogenic differentiation of dental pulp stem cells (DPSCs), this type of enrichment requires specific expertise and cannot be performed by a clinician [28]. Whereas in the case of the ALAD-PDT protocol, it can be applied directly by the clinician during dental surgery. A further advantage for clinicians and patients is the time of ALAD-PDT application, which requires only 45 min for gel incubation and 7 min of irradiation compared to other photodynamic protocols that require 2–3 h incubation time and 20–25 min for irradiation [35,36]. Altogether, the results of this preliminary study may suggest a possible combination of two different techniques, a photodynamic protocol and a curved cortical membrane, commonly applied in dentistry fields for different purposes, to improve the biological properties of the lamina cortical membranes applied during GBR procedures, in promoting the bone healing and remodeling processes. This in vitro study might be a base for further in vivo experimentations, and it opens up a combination of two techniques until now used in the dentistry field for different purposes, and clinicians can easily apply that.

## 5. Conclusions

In conclusion, the adjunctive application of ALAD-PDT, an antibacterial photodynamic protocol, to oral osteoblasts cultured on a cortical lamina provided a higher cellular proliferation and adhesion and increased matrix bone deposition.

## Data Availability

MDPI Research Data Policies.

## References

[1] Lan M., Zhao S., Liu W., Lee C.S., Zhang W., Wang P. (2019). Photosensitizers for Photodynamic Therapy. Adv. Healthc. Mater..

[2] Kwiatkowski S., Knap B., Przystupski D., Saczko J., Kędzierska E., Knap-Czop K., Kotlińska J., Michel O., Kotowski K., Kulbacka J. (2018). Photodynamic Therapy—Mechanisms, Photosensitizers and Combinations. Biomed. Pharmacother..

[3] Kou J., Dou D., Yang L. (2017). Porphyrin Photosensitizers in Photodynamic Therapy and Its Applications. Oncotarget.

[4] Stájer A., Kajári S., Gajdács M., Musah-Eroje A., Baráth Z. (2020). Utility of Photodynamic Therapy in Dentistry: Current Concepts. Dent. J..

[5] Qian Y., Wang J., Bu W., Zhu X., Zhang P., Zhu Y., Fan X., Wang C. (2022). Targeted Implementation Strategies of Precise Photodynamic Therapy Based on Clinical and Technical Demands. Biomater. Sci..

[6] Fukuhara H., Inoue K., Kurabayashi A., Furihata M., Fujita H., Utsumi K., Sasaki J., Shuin T. (2013). The Inhibition of Ferrochelatase Enhances 5-Aminolevulinic Acid-Based Photodynamic Action for Prostate Cancer. Photodiagnosis Photodyn. Ther..

[7] Ohgari Y., Nakayasu Y., Kitajima S., Sawamoto M., Mori H., Shimokawa O., Matsui H., Taketani S. (2005). Mechanisms Involved in Delta-Aminolevulinic Acid (ALA)-Induced Photosensitivity of Tumor Cells: Relation of Ferrochelatase and Uptake of ALA to the Accumulation of Protoporphyrin. Biochem. Pharmacol..

[8] Sachar M., Anderson K.E., Ma X. (2016). Protoporphyrin IX: The Good, the Bad, and the Ugly. J. Pharmacol. Exp. Ther..

[9] Li X., Guo H., Tian Q., Zheng G., Hu Y., Fu Y., Tan H. (2013). Effects of 5-Aminolevulinic Acid-Mediated Photodynamic Therapy on Antibiotic-Resistant Staphylococcal Biofilm: An in Vitro Study. J. Surg. Res..

[10] Nakai Y., Tatsumi Y., Miyake M., Anai S., Kuwada M., Onishi S., Chihara Y., Tanaka N., Hirao Y., Fujimoto K. (2016). Expression of Ferrochelatase Has a Strong Correlation in Protoporphyrin IX Accumulation with Photodynamic Detection of Bladder Cancer. Photodiagnosis Photodyn. Ther..

[11] Radunović M., Petrini M., Vlajic T., Iezzi G., di Lodovico S., Piattelli A., D’Ercole S. (2020). Effects of a Novel Gel Containing 5-Aminolevulinic Acid and Red LED against Bacteria Involved in Peri-Implantitis and Other Oral Infections. J. Photochem. Photobiol. B.

[12] Petrini M., di Lodovico S., Iezzi G., Cellini L., Tripodi D., Piattelli A., D’ercole S. (2022). Photodynamic Antibiofilm and Antibacterial Activity of a New Gel with 5-Aminolevulinic Acid on Infected Titanium Surfaces. Biomedicines.

[13] di Lodovico S., Diban F., di Fermo P., Petrini M., Fontana A., di Giulio M., Piattelli A., D’ercole S., Cellini L. (2022). Antimicrobial Combined Action of Graphene Oxide and Light Emitting Diodes for Chronic Wound Management. Int. J. Mol. Sci..

[14] Rossi R., Rispoli L., Lopez M.A., Netti A., Petrini M., Piattelli A. (2022). Photodynamic Therapy by Mean of 5-Aminolevulinic Acid for the Management of Periodontitis and Peri-Implantitis: A Retrospective Analysis of 20 Patients. Antibiotics.

[15] D’Ercole S., Carlesi T., Dotta T.C., Pierfelice T.V., D’Amico E., Tripodi D., Iezzi G., Piattelli A., Petrini M. (2022). 5-Aminolevulinic Acid and Red Led in Endodontics: A Narrative Review and Case Report. Gels.

[16] Petrini M., Pierfelice T.V., D’amico E., Carlesi T., Iezzi G., D’arcangelo C., di Lodovico S., Piattelli A., D’ercole S. (2022). Comparison between Single and Multi-LED Emitters for Photodynamic Therapy: An In Vitro Study on Enterococcus Faecalis and Human Gingival Fibroblasts. Int. J. Environ. Res. Public Health.

[17] Pierfelice T.V., D’Amico E., Iezzi G., Petrini M., Schiavone V., Santalucia M., Pandolfi A., D’Arcangelo C., Piattelli A., di Pietro N. (2022). Effect of a 5-Aminolevulinic Acid Gel and 660 Nm Red LED Light on Human Oral Osteoblasts: A Preliminary in Vitro Study. Lasers Med. Sci..

[18] Pierfelice T.V., D’Amico E., Petrini M., Pandolfi A., D’Arcangelo C., di Pietro N., Piattelli A., Iezzi G. (2022). The Effects of 5% 5-Aminolevulinic Acid Gel and Red Light (ALAD-PDT) on Human Fibroblasts and Osteoblasts. Gels.

[19] Elgali I., Omar O., Dahlin C., Thomsen P. (2017). Guided Bone Regeneration: Materials and Biological Mechanisms Revisited. Eur. J. Oral Sci..

[20] Fontana F., Maschera E., Rocchietta I., Simion M. (2011). Clinical Classification of Complications in Guided Bone Regeneration Procedures by Means of a Non Resorbable Membrane. Int. J. Periodontics Restor. Dent..

[21] Becerra J., Rodriguez M., Leal D., Noris-Suarez K., Gonzalez G. (2022). Chitosan-Collagen-Hydroxyapatite Membranes for Tissue Engineering. J. Mater. Sci. Mater. Med..

[22] Aziz B., Aziz I., Khurshid A., Raoufi E., Esfahani F.N., Jalilian Z., Mozafari M.R., Taghavi E., Ikram M. (2023). An Overview of Potential Natural Photosensitizers in Cancer Photodynamic Therapy. Biomedicines.

[23] Gallardo-Villagrán M., Leger D.Y., Liagre B., Therrien B. (2019). Photosensitizers Used in the Photodynamic Therapy of Rheumatoid Arthritis. Int. J. Mol. Sci..

[24] Gholami L., Shahabi S., Jazaeri M., Hadilou M., Fekrazad R. (2023). Clinical Applications of Antimicrobial Photodynamic Therapy in Dentistry. Front. Microbiol..

[25] Ramos U.D., Suaid F.A., Wikesjö U.M.E., Susin C., Taba M., Novaes A.B. (2017). Comparison between Two Antimicrobial Protocols with or without Guided Bone Regeneration in the Treatment of Peri-Implantitis. A Histomorphometric Study in Dogs. Clin. Oral Implant. Res..

[26] Garcia de Carvalho G., Sanchez-Puetate J.C., Casalle N., Marcantonio Junior E., Leal Zandim-Barcelos D. (2020). Antimicrobial Photodynamic Therapy Associated with Bone Regeneration for Peri-Implantitis Treatment: A Case Report. Photodiagnosis Photodyn. Ther..

[27] Liu Q., Wang L. (2021). T-Test and ANOVA for Data with Ceiling and/or Floor Effects. Behav. Res. Methods.

[28] di Carlo R., Zara S., Ventrella A., Siani G., da Ros T., Iezzi G., Cataldi A., Fontana A. (2019). Covalent Decoration of Cortical Membranes with Graphene Oxide as a Substrate for Dental Pulp Stem Cells. Nanomaterials.

[29] Wachtel H., Fickl S., Hinze M., Bolz W., Thalmair T. (2013). The Bone Lamina Technique: A Novel Approach for Lateral Ridge Augmentation--a Case Series. Int. J. Periodontics Restor. Dent..

[30] O’Brien F.J., Harley B.A., Yannas I.V., Gibson L.J. (2005). The Effect of Pore Size on Cell Adhesion in Collagen-GAG Scaffolds. Biomaterials.

[31] Chang H.-I., Wang Y. (2011). Cell responses to surface and architecture of tissue engineering scaffolds. Regenerative Medicine and Tissue Engineering—Cells and Biomaterials.

[32] Wu Z., Zhong J., Yu Y., Rong M., Yang T. (2021). A Rapid and Convenient Approach to Construct Porous Collagen Membranes via Bioskiving and Sonication-Feasible for Mineralization to Induce Bone Regeneration. Front. Bioeng. Biotechnol..

[33] Lauritano D., Moreo G., Palmieri A., della Vella F., Petruzzi M., Botticelli D., Carinci F. (2022). Photodynamic Therapy Using 5-Aminolevulinic Acid (Ala) for the Treatment of Chronic Periodontitis: A Prospective Case Series. Appl. Sci..

[34] Azaripour A., Azaripour M., Willershausen I., van Noorden C.J.F., Willershausen B. (2018). Photodynamic Therapy Has No Adverse Effects In Vitro on Human Gingival Fibroblasts and Osteoblasts. Clin. Lab..

[35] Ou J., Gao Y., Li H., Ling T., Xie X. (2022). Application of 5-Aminolevulinic Acid-Mediated Waterlase-Assisted Photodynamic Therapy in the Treatment of Oral Leukoplakia. Sci. Rep..

[36] Jin J., Zhang Y., Zhiyue L. (2019). Successful Treatment of Oral Human Papilloma by Local Injection 5-Aminolevulinic Acid-Mediated Photodynamic Therapy: A Case Report. Photodiagnosis Photodyn. Ther..

